# Scattering Analysis and Efficiency Optimization of Dielectric Pancharatnam–Berry-Phase Metasurfaces

**DOI:** 10.3390/nano11030586

**Published:** 2021-02-26

**Authors:** Chen-Yi Yu, Qiu-Chun Zeng, Chih-Jen Yu, Chien-Yuan Han, Chih-Ming Wang

**Affiliations:** 1 Department of Optics and Photonics, National Central University, Taoyuan 32001, Taiwan; 108226032@cc.ncu.edu.tw (C.-Y.Y.); xczaqs85@gmail.com (Q.-C.Z.); 2 Graduate Institute of Electro-Optical Engineering, Chang Gung University, Taoyuan 333, Taiwan; cjyu@mail.cgu.edu.tw; 3 Department of Electro Optical Engineering, National United University, Miaoli 36063, Taiwan; cyhan@nuu.edu.tw

**Keywords:** PB-phase, metasurface, polarization

## Abstract

In this study, the phase modulation ability of a dielectric Pancharatnam–Berry (PB) phase metasurface, consisting of nanofins, is theoretically analyzed. It is generally considered that the optical thickness of the unit cell of a PB-phase metasurface is *λ*/2, i.e., a half-waveplate for polarization conversion. It is found that the *λ*/2 is not essential for achieving a full 2*π* modulation. Nevertheless, a *λ*/2 thickness is still needed for a high polarization conversion efficiency. Moreover, a gradient phase metasurface is designed. With the help of the particle swarm optimization (PSO) method, the wavefront errors of the gradient phase metasurface are reduced by fine-tuning the rotation angle of the nanofins. The diffraction efficiency of the gradient phase metasurface is thus improved from 73.4% to 87.3%. This design rule can be utilized to optimize the efficiency of phase-type meta-devices, such as meta-deflectors and metalenses.

## 1. Introduction

Recently, Pancharatnam–Berry (PB) metasurfaces [1,2], based on design concept regarding the geometric phase, have attracted intense attention due to their strong capabilities in controlling circular-polarized (CP) waves [3]. We consider the case of two identical metasurface unit cells illuminated by the same CP light, where the second one is rotated by an angle *θ_p_* with respect to the first one. The spin-flipped components of the waves, scattered by the two unit cells, will differ only with a phase factor *e^i2^^θp^*. This phase difference, termed as the PB-phase, is independent of the frequency. This dispersion-less phase modulation ability allows the PB-phase metasurface to control the phase much easier compared to other resonance-based metasurfaces. However, the unavoidable Ohmic heat dissipation loss of metals significantly degrades the performances of metasurfaces made by plasmonic structures [4]. To address the heat dissipation loss of metals, dielectric metasurfaces are proposed [5,6]. Low-loss dielectric resonators can also support both electric and magnetic resonances with mechanism governed by the Mie resonances [7]. For the lossless case, a single electric resonator exhibits a Lorentz-type response with a maximum phase variation of *π* and with a scattering amplitude tightly linked with the phase variation. Nevertheless, with an optically thick-enough nanofin with optical anisotropy, the strict *π* limit is no longer applicable on the single electric resonator due to the magnetic dipole resonance arisen from the circulating displacement currents within the nanofin. In general, by overlapping the electric and magnetic resonances in frequency via adjusting the resonator’s geometry, it is possible to achieve a phase variation covering the entire 2*π* range with high transmission [8,9].

As a 100% efficient PB metasurface in transmission geometry, a perfectly transparent unit cell is not sufficient. According to the PB-phase concept, a polarization converter is also needed [10]. The polarization converter is usually a half-wave plate (HWP) [11]. Therefore, utilizing the anisotropy of the unit cell to obtain a HWP is a common strategy for achieving high-efficiency metasurfaces.

Here, we consider PB-phase metasurfaces that operate for the CP. We consider a simple shape of the antenna, a nanofin. The nanofin can be regarded as a one-dimensional dipole oscillator, where the rotation angles of the antennas impart an initial phase difference to the outgoing circularly polarized light. This mechanism of the phase modulation is not based on the resonance condition. Therefore, compared to other resonance-based antennas, PB phase metasurfaces have a broader range of operating wavelengths. In this article, we would like to analyze how the thickness of the nanofin affects the modulation phase of CP and propose a high-efficiency gradient phase metasurface based on optimized geometric parameters of the nanofin.

## 2. Simulation of Phase Modulation of PB-phase Metasurfaces

Imagine an x-polarized normally incident light impinging onto a nanofin with a rotation angle (respect to the *x*-axis) of *θ_p_*. The output polarization state is elliptically polarized light and can be decomposed to two cross-linear polarization states, as shown on the left-hand side of Equation (1). The amplitude parts of the linear polarization states are decomposed to two cross- and circular-polarization states [12]:

(1) $$\left[\begin{array}{c} \left|E_{x}\right|e^{i\delta_{x}}\\ \left|E_{y}\right|e^{i\delta_{y}} \end{array}\right]=\left[\begin{array}{c} e^{i\delta_{x}}(a_{x}\hat{R}+b_{x}\hat{L})\\ e^{i\delta_{y}}(a_{y}\hat{R}+b_{y}\hat{L}) \end{array}\right]=\left[\begin{array}{c} \frac{1}{\sqrt{2}}\left|E_{x}\right|e^{i\delta_{x}}(\hat{R}+\hat{L})\\ \frac{1}{\sqrt{2}}\left|E_{y}\right|e^{i\delta_{y}}(\hat{R}-\hat{L}) \end{array}\right]$$

where $\hat{R}$ and $\hat{L}$ is the normalized Jones vector of ideal right-handed circularly polarized (RCP) and left-handed circularly polarized (LCP), respectively. The output phasor of the RCP, *E_R_*, can be obtained by retrieving the RCP components in the *E_x_* and *E_y_*. The phasor of the LCP, *E_L_*, can be similarly obtained.

(2) $$E_{R}=a_{x}e^{i\delta_{x}}+a_{y}e^{i\delta_{y}}=\left|E_{R}\right|e^{i\delta_{R}}=\frac{1}{\sqrt{2}}\left(\left|E_{x}\right|e^{i\delta_{x}}+i\left|E_{y}\right|e^{i\delta_{y}}\right)$$

(3) $$E_{L}=b_{x}e^{i\delta_{x}}+b_{y}e^{i\delta_{y}}=\left|E_{L}\right|e^{i\delta_{L}}=\frac{1}{\sqrt{2}}\left(\left|E_{x}\right|e^{i\delta_{x}}-i\left|E_{y}\right|e^{i\delta_{y}}\right)$$

**Figure 1:**
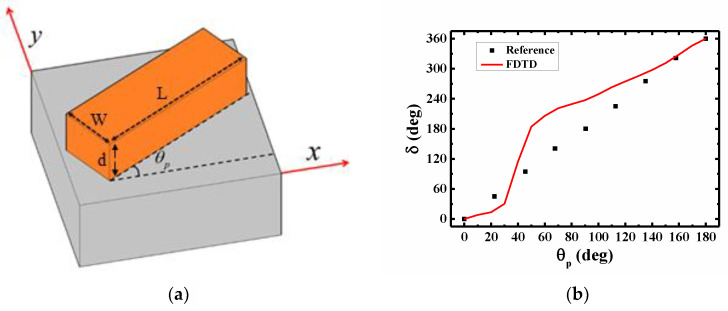
(**a**) Schematic of the unit cell of the PB phase metasurface consisting of a GaN nanofin on an Al_2_O_3_ substrate. L, W and d indicate the length, width, and thickness of the nanofin, respectively. (**b**) The overall phase modulation through a periodic nanofin with a geometric parameters of L = 330 nm, W = 100 nm, and d = 600 nm, respectively. The red line indicates our simulation result based on Equation (3). The solid square symbol represents the FDTD simulation results from [15].

According to Equations (2) and (3), we can retrieve the overall phase modulation, *δ*, of the incident light passing through the metasurface, which are GaN nanofins on an Al_2_O_3_ substrate. *δ_R_* and *δ_L_* indicate the overall phase modulation for the output beam with a polarization state of RCP and LCP, respectively. The schematic of the investigated structure is shown in Figure 1a. L, W and d indicate the length, width, and height of the nanofin, respectively. The optical properties of the GaN nanofin on an Al_2_O_3_ substrate are simulated by using the Finite-Difference Time-Domain (FDTD) method, a commercial software package FullWave by RSoft-SYNOPSYS. Figure 1b shows the phase modulation as a function of *θ_p_* for normally incident light with RCP. The geometric parameters of the nanofin are L = 330 nm, W = 100 nm, and d = 600 nm, respectively. The period of the unit cell is 330 nm. The operation wavelength is set to be 633 nm. The dielectric constants of the GaN and Al_2_O_3_ are taken from [13,14]. Periodic and perfectly matched layer (PML) boundary conditions are used in the transverse direction and vertical direction, respectively. The total length in the z-direction is 10 μm and the nanofin/Al_2_O_3_ interface is set at z = 0 μm. The solid red line shown in Figure 1b indicates our simulation result based on Equation (3) which is the overall phase modulation. The black squares represent the results taken from the reference [15], which was simulated by using the Finite Element Method. They simulated the phase as the input polarization is fully converted to its cross-polarization state. The original phase reference of reference 15 is *θ_p_* = 22.5°. For comparison, we shift the phase reference from *θ_p_* = 22.5° to *θ_p_* = 0°. Here, we consider the phase modulation of the total transmission field of incident light with x-polarization but not only the geometric phase as LCR converts to RCP. Therefore, it shows a nonlinear phase modulation curve as a function of the *θ_p_* as the input polarization is not fully converted to its cross polarization state. Moreover, the nanofin that is used has a *θ_p_* of 0° as a phase reference. Therefore, there is a constant shift of the two overall phase modulation curves.

The overall phase modulation of a PB-phase metasurface is the combination of the dynamic phase and geometric phase. The dynamic phase relies on the optical path length (OPL), which is the product of the thickness (d) and the refractive index (n) of an optical element and is independent of the orientation angle of the nanofin, *θ_p_*. The geometric phase is the inherent phase modulation of the PB phase, owing to its rotational symmetry. The magnitude of the geometric-phase shift is 2*θ_p_*.

**Figure 2:**
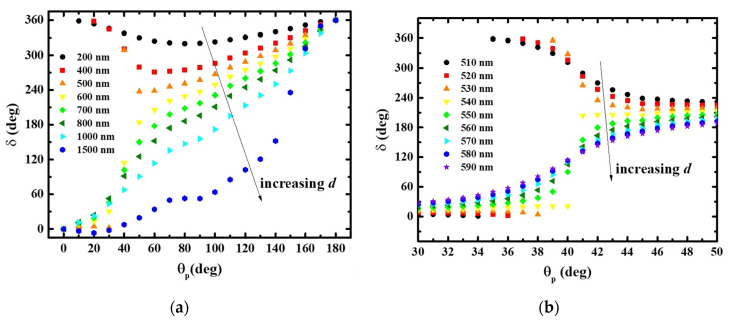
(**a**) Overall phase modulation (*δ*) of a typical dielectric PB-phase metasurface as a function of *θ_p_*. The black, red, orange, yellow, green, olive, cyan, and blue colors indicate the thickness of 200 nm, 400 nm, 500 nm, 600 nm, 700 nm, 800 nm, 1000 nm, and 1500 nm, respectively. (**b**) Overall phase modulation (*δ*) as a function of *θ_p_*. The black, red, orange, yellow, green, olive, cyan, blue, and violet represent the thickness of 510 nm to 590 nm with a step of 10 nm.

Here, we keep the same length and width of the nanofin. The phase modulation under a series of different thickness are simulated and shown in Figure 2a. Usually, for off-resonance conditions, the performance of a metallic nanofin design is not sensitive to the width and height [16,17]. However, the dynamic phase of a dielectric antenna significantly is affected by the thickness. Generally, we can see two trends in Figure 2a. For a very thin thickness, the overall phase is dominated by the geometric phase. As *θ_p_* increases, the overall phase will slightly decrease from 360° for *θ_p_* = 0^o^. It shows a minimum at *θ_p_* = 90° and then increases back to 360° for *θ_p_* =180° due to the symmetry. Thus, we cannot achieve 2*π* phase modulation. For a relatively thick thickness, the overall phase can increase from 0° for *θ_p_* = 0° to 360° for *θ_p_* = 180°. As shown in Figure 2a, there is a critical or transition thickness between d = 500~600 nm. The overall phase trend is split toward 0° and 360° for *θ_p_* = 180° and the transition occurs for *θ_p_* close to 45°. For clarity, we again simulate the overall phase as a function of thickness and *θ_p_* as shown in Figure 2b. The geometric parameters of the nanofin are still 300 nm × 100 nm with a pitch of 330 nm. It can be seen that the critical thickness is 540 nm. It is shown that 2*π* overall phase modulation is achieved for d > 540 nm. A linear overall phase modulation as a function of *θ_p_* can be obtained for d = 1000 nm. It was reposted that the complete 2*π* phase control of the PB-phase mechanism is due to the electromagnetic resonance and the strong confinement of the incident wave inside the dielectric nanofins [18].

## 3. Results and Discussion

### 3.1. Analysis of the Critical Thickness and Peak of the Overall Phase Modulation

**Figure 3:**
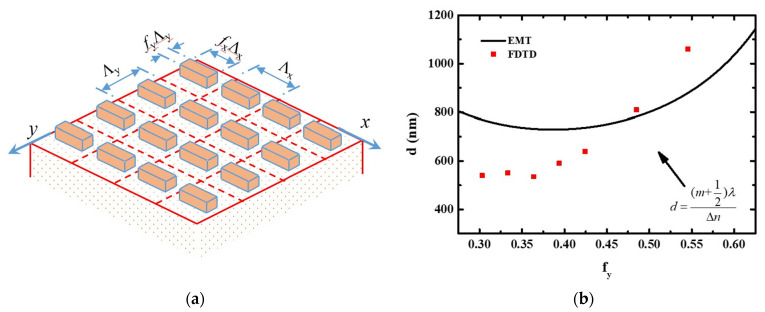
(**a**) Schematic of the periodic dielectric nanofin array on an Al_2_O_3_ substrate. (**b**) FDTD simulated critical thickness for full 2*π* modulation as a function of *f_y_* (red square). The 2D EMT is represented by solid black line. The Δ*n* for periodic GaN nanofin array is calculated at a constant wavelength-to-period ratio of 0.633/0.33.

Figure 3 shows the schematic of the periodic dielectric nanofin array on an Al_2_O_3_ substrate. The nanofin antenna can be considered as a two-dimensional dielectric grating, whose effective refractive index can be analyzed by using effective medium theory (EMT). The filling factor *f* is defined by the ratio of the ridge width to the period Λ. The subscript, *x* and *y*, indicates the *x*- and *y*- directions. Therefore, the two grating vectors of the nanofin array can be denoted by *K_x_* = 2*π*/$\Lambda_{x}$ and *K_y_* = 2*π*/$\Lambda_{y}$. The nanofin is two-folded symmetric around the *z*-axis and behaves as a formed-birefringent anisotropic medium. Here, we attempt to obtain the effective indices of the nanofin array based on the second-order 1D EMT theory. The effective index for a propagating wave along the *z*-axis with an electric field polarized along the *x*-axis is *n_x_*. Similarly, the effective index for a propagating wave along the *z*-axis with y-polarization is *n_y_*. First, we reduce the 2D nanofin array to be an effective 1D grating with a grating vector of *K_y_* by using 1D EMT theory [19,20]. Assume the incident light is x-polarized. The zero-order approximation of the effective index of the metasurface can be expressed as the following:

(4) $$\varepsilon_{x,E∥K_{x},1D}^{\left(0\right)}={\left(n_{x,E∥K_{x},1D}^{\left(0\right)}\right)}^{2}=\frac{\varepsilon_{A}\varepsilon_{B}}{f_{x}\varepsilon_{A}+\;(1-f_{x})\;\varepsilon_{B}\;}$$

(5) $$\varepsilon_{y,\;E⊥K_{x},\;1D}^{\left(0\right)}={\left(n_{y,\;E⊥K_{x},\;1D}^{\left(0\right)}\right)}^{2}=\varepsilon_{A}(1-f_{x})+\varepsilon_{B}f_{x}$$

where $\varepsilon_{A}$ and $\varepsilon_{B}$ are the permittivity of the host material and nanofin material, respectively. In this case, the $\varepsilon_{A}$ and $\varepsilon_{B}$ are the permittivity of air and GaN, respectively. Since the period-to-wavelength ratio is small, the displacement vector and the electric field are approximately constant across the unit cell. For zero-order approximation, the effective index is independent of the ratio of the grating period to wavelength, Λ*_g_*/*λ*. In this paper, the second-order solutions are applied as shown:

(6) $$\varepsilon_{x,\;E∥K_{x},\;1D}^{\left(2\right)}=\;\varepsilon_{x,\;E∥K_{x},\;1D}^{\left(0\right)}+\frac{{\;\pi }^{2}}{3}f_{x}^{2}{\left(1-f_{x}\right)}^{2}\left(\frac{\Lambda_{x}}{\lambda }\right){\left(\frac{1}{\;\varepsilon_{A}}-\frac{1}{\;\varepsilon_{B}}\right)}^{2}{\left(\varepsilon_{x,\;E∥K_{x},\;1D}^{\left(0\right)}\right)}^{3}\cdot \varepsilon_{y,\;E⊥K_{x},\;1D}^{\left(0\right)}$$

By using Equation (6), we can reduce the nanofin array to be an effect 1D grating with an effective permittivity calculated by Equation (6) and a host material of air. Then, again utilize the 1D EMT theory to reduce the effective 1D grating to an effective homogeneous medium. Again, the zero-order approximation is:

(7) $$\varepsilon_{x,\;E⊥K_{y},\;1D}^{\left(0\right)}={\left(n_{x,\;E⊥K_{y},\;2D}^{\left(0\right)}\right)}^{2}=\varepsilon_{A}(1-f_{y})+\varepsilon_{x,\;E∥K_{x},\;1D}^{\left(2\right)}f_{y}$$

Note that the x-polarized light is perpendicular to *K_y_* and the $\varepsilon_{B}$ now is the effective permittivity of 1D GaN grating but not the permittivity of GaN. The second-order approximation for 2D nanofin is:

(8) $$\varepsilon_{x,\;E⊥K_{y},\;2D}^{\left(2\right)}=\;\varepsilon_{x,\;E⊥K_{y},\;2D}^{\left(0\right)}+\frac{{\;\pi }^{2}}{3}f_{y}^{2}{\left(1-f_{y}\right)}^{2}\left(\frac{\Lambda_{y}}{\lambda }\right){\left(\varepsilon_{x,\;E⊥K_{y},\;2D}^{\left(0\right)}-\varepsilon_{A}\right)}^{2}$$

The effective refractive index for y-polarization, *n_y_*, can also be obtained by a similar procedure. The most commonly used EMTs are the Maxwell–Garnett Equation [21,22], Bruggeman’s model [23], and the Lorentz–Lorentz model [24]. All of these have been demonstrated to be in good agreement with other rigorous simulation methods, such as Rigorous coupled-wave analysis (RCWA) and FDTD.

In order to find the relation between the critical thickness and the effective index of the nanofin array. The half-wavelength thickness calculated by EMT and critical thickness simulated by FDTD method as a function of *f_y_* is shown in Figure 3b. As mentioned, the critical thickness, which is denoted by square symbols, is defined as the minimum thickness for achieving a full 2*π* modulation simulated by FDTD method. The solid line indicates the half-wave thickness calculated by using the EMT and the following equation:

(9) $$\Delta n\;d=(m+\frac{1}{2})\lambda$$

where Δ*n = *(*n_y_ − n_x_*)** and *m* = 0. It is well-known that the input CP state will turn to an orthogonal CP as it passes through a PB phase metasurface. Therefore, besides providing the geometric phase, the PB phase metasurface also plays an important role in polarization conversion. For the point of view of a waveplate, for a fixed wavelength and Δ*n*, the polarization conversion efficiency is a function of its thickness. The square symbols indicate the critical thickness simulated by using FDTD. As mentioned, the *f_x_* is 10/11. The anisotropic effect is similar to a one-dimensional (1D) grating under such a large *f_x_*. According to the EMT, the maximum Δ*n* for 1D grating occurs at a filling factor of 0.5. The 2D EMT shows a similar trend. There is a maximum Δ*n* as *f_y_* = 0.4. Δ*n* equal to zero for *f_y_* = 0 and close to zero for *f_y_* = 1. As *f_y_* away from 0.4, the optical anisotropic effect is less significant, i.e., a small Δ*n*. Therefore, a relatively thick d is required. However, the critical thickness simulated by FDTD is not the optimized thickness, i.e., the *λ*/2 thickness. The critical thickness is the minimum thickness for achieving 2*π* modulation. However, under the critical thickness, the polarization conversion efficiency is not sufficient. The optimized thickness simulated by FDTD for highest polarization conversion efficiency is 1000 nm. Simply speaking, for achieving 2*π* modulation, *λ*/2 thickness is not essential. Nevertheless, *λ*/2 is the optimized thickness for a high conversion efficiency Moreover, this thickness is the corresponding *λ*/2 thickness which is about 250 nm thicker than that predicted by the EMT. We think the discrepancy between the FDTD and EMT is because the EMT ignores the change of the period and the filling factor caused by the rotation of the nanofin.

**Figure 4:**
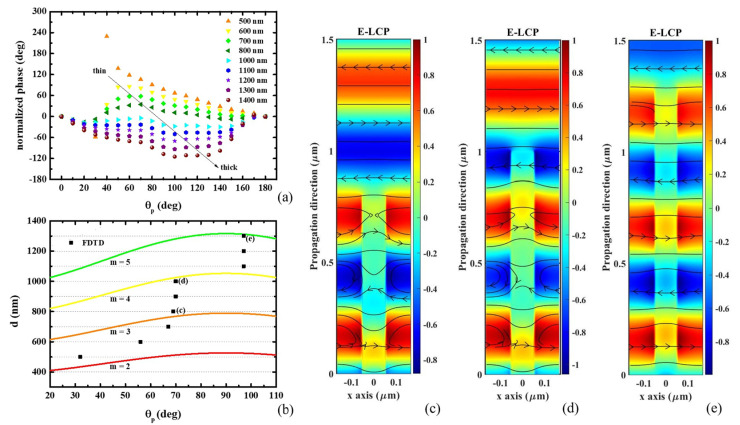
(**a**) Normalized overall phase modulation (*δ*) as a function of *θ_p_*, *δ’ (θ_p_)*. (**b**) Thickness at the peak of the normalized overall phase modulation (*δ*) as a function of *θ_p_*. (**c**) The E-field distribution and the magnetic line for d = 800 nm. (**d**) The E-field distribution and the magnetic line for d = 1000 nm. (**e**) The E-field distribution and the magnetic line for d = 1300 nm.

For observing the nonlinearity of the overall phase modulation curve as a function of *θ_p_*, we normalize the overall phase modulation as a function of *θ_p_, δ(θ_p_)*, by an ideal *δ*-*θ_p_* function, *δ* = *2θ_p_*. As shown in Figure 4a, the normalized *δ*, *δ’*, shows a peak at *θ_p_* = 55° for a d = 600 nm. The peak shifts to a larger *θ_p_* for an increasing d. The peak transforms to a dip as *θ_p_* > 90°. When the inclined angle between the x-polarization and the long-axis of the nanofin is zero, the effective refractive index, *n_eff, x_*, is *n_x_*. For *θ_p_* = 90°, *n_eff, x_* is equal to *n_y_*. For a rotating *θ_p_*, the effective refractive index for the x-polarization can be calculated by using the following equation:

(10) $$\frac{1}{n_{eff,\;x}^{2}(\theta_{p})}=\frac{\cos^{2}\theta_{p}}{n_{x}^{2}}+\frac{\sin^{2}\theta_{p}}{n_{y}^{2}}$$

It is found that the peak/dip of *δ’*-*θ_p_* relation coincides with the high reflection condition of a single-layer thin-film. The high reflection condition is:

(11) $$2\times n_{eff,\;x}^{}(\theta_{p})\times d=m\lambda$$

where *m* is the number of the standing waves. Figure 4b shows the thickness for the high reflection condition calculated by Equation (11). The red, orange, yellow, and green lines indicate the *m* = 2, 3, 4, and 5, respectively. The E-field distribution and the magnetic line for d = 500 nm, 700 nm, 1000 nm are shown in Figure 4c–e, respectively. The E-field distribution of the LCP component shows the standing wave resonance in the effective thin film, i.e., the combination of air and nanofin. The number of the standing wave for d = 800 nm, 1000 nm, 1300 nm are *m* = 3, 4, 5, respectively, which has a good agreement with the calculated results from using the EMT. Moreover, from the vertex of the magnetic line, one can observe the polarization conversion during propagating in the nanofin.

For x-polarized light, the effective index, *n_eff, x_*, gradually decreases from 1.6138 to 1.2023 as *θ_p_* increases from 0° to 90°. By fulfilling the condition of Equation (11), when the phase peak shifts to larger *θ_p_*, the d increases as shown in Figure 4a,b. Furthermore, the peak transforms to a dip as it crosses over the transmission point at *θ_p_* = 90°. Although it is not shown here, the RCP component shows a complementary behavior. The effective index, *n_eff, y_*, gradually increases from 1.2023 to 1.6138 as *θ_p_* increases from 0° to 90°. Consequently, the phase dip shifts to small *θ_p_* as d increases. As the peak and dip coincides at *θ_p_* = 90°, by choosing an appropriate d, the overall phase modulation is almost linear to *θ_p_*. A linear phase modulation curve is more convenient for practical applications.

### 3.2. Phase Influence Caused by Adjacent Unit Cells

**Figure 5:**
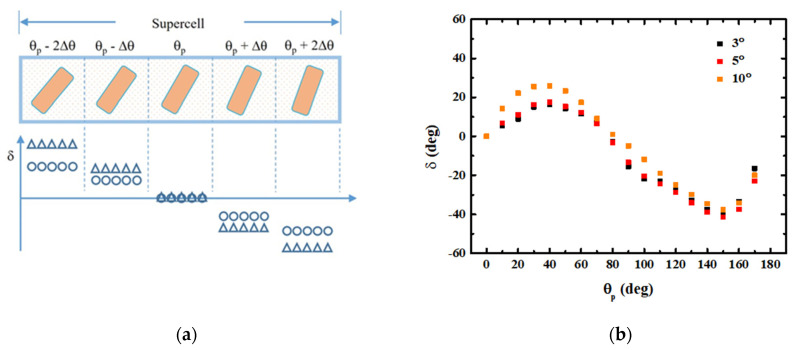
(**a**) Schematic of the considered supercell. The supercell consists of five unit cells. The rotation angle of the adjacent unit cell is ±Δθ respect to its neighbor unit cell. (**b**) Overall phase at center unit cell for Δθ = 3°, 5° and 10°, respectively.

The previous results are simulated under the assumption that all the unit cells are identical and periodical. For this kind of periodic boundary conditions, the phase modulation (at an arbitrary x- and y- position) of a PB-phase unit cell is uniform. The non-uniform scattering term of E_x_ and E_y_ components (the eigenstates of circular polarization) is compensated by the diffracted/scattering beam from the adjacent and identical unit cells. However, for a practical application, the adjacent unit cell is not identical. Consequently, the non-uniform components cannot be compensated, which leads to a serious scattering loss. As a result, the overall efficiency of metalenses and gradient surfaces is currently lower than conventional refractive-type device. In this part, we can consider a unit cell sandwiched by unit cells along the x-direction. In the y-direction, the unit cells are identical. For convenience, the central unit cell is named CU, while the two adjacent unit cells at the left-hand-side and right-hand-side are called LU and RU, respectively. The rotation angle of LU and RU are ($\theta_{p}$ + Δθ) and ($\theta_{p}$ − Δθ), respectively. The schematic of the considered supercell is shown in Figure 5a. The triangle and circle symbols indicate corresponding phase for a large Δθ and a small Δθ, respectively. The overall phase at CU for different Δθ as a function of $\theta_{p}$ is shown in Figure 5b. It can be seen that as the adjacent unit cell is not identical to the CU, the overall δ of the CU is different from that of the periodic case. In addition, generally, a larger Δθ results in a larger phase difference, compared to a periodic case shown in Figure 1b. Therefore, it seems to us that the induced phase difference is not only arisen from the electromagnetic coupling of the adjacent unit cell, but also the phasor evolution during propagation. This result shows that the adjacent scattering is important for designing high-efficiency meta-devices. The scattering losses from the non-uniform phase modulation should be eliminated by appropriately designing the supercell.

### 3.3. Demonstration of Phase Optimization with Gradient Surface Metasurface

**Figure 6:**
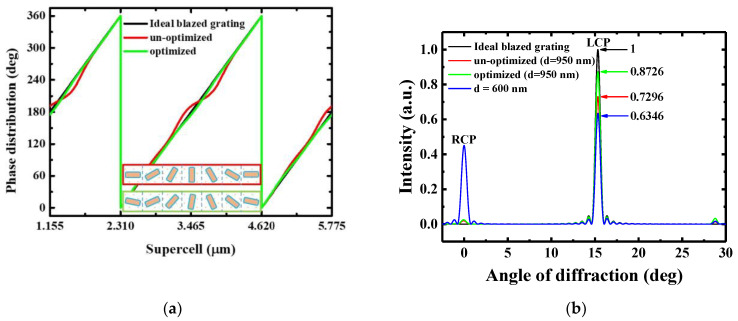
(**a**) Phase distribution within one supercell. The black, green, and red lines indicate the ideal blazed grating, un-optimized gradient surface metasurface, and optimized gradient surface metasurface, respectively. (**b**) Far-field diffraction efficiency of the ideal blazed grating (black), un-optimized gradient surface metasurface (green), and optimized gradient surface metasurface (red).

In this part, we utilize a gradient surface metasurface [25] as a demonstration of phase optimization. Basically, the gradient surface metasurface is similar to a blazed grating and is also similar to the outer ring of a metalens. Theoretically, the phase distribution constructed by using a metasurface can be a continuous gray-level one in a one-step lithography process. This means the theoretical diffraction efficiency can be close to 100% as the Fresnel reflection loss can be ignored. In contrast, the diffraction efficiency of an eight-level phase grating is 92%. Furthermore, a three-step lithography process is required. Here we use a gradient surface metasurface with a supercell consisting of 7 unit cells. Figure 6a shows the phase distribution within one supercell for ideal blazed grating, un-optimized gradient surface metasurface, and optimized gradient surface metasurface. The phase distribution is recorded for the incident light away from the metasurface with a distance of 3 μm. For ideal blazed grating, the phase linearly increases from 0 to 2*π*. For the un-optimized meatasurface, the rotation angle of the un-optimized metasurface is directly taken from Figure 2a. The phase distribution vibrates owing to the influence of the adjacent unit cells. The maximum phase difference as compared to the ideal one is 16.8°. This oscillation in phase leads to scattering loss. In free-space, the direction of energy flow is normal to the wavefront, i.e., $\vec{S}∥\vec{k}$, where $\vec{S}$ and $\vec{k}$ is the Poynting vector and wave-vector, respectively. Therefore, to achieve a high diffraction efficiency, a continuous and smooth phase profile is essential. Here, the phase optimization is done by using particle swarm optimization method [26,27]. The merit function is the deviation of the under-optimized phase to the ideal phase profile, i.e., the wavefront error. The optimization variables are the rotation angles of the nanofins in the supercell, whereas as is shown in Figure 6a, the phase distribution becomes close to the ideal one after PSO optimization. The schematic of the arrangement of the nanofins with and without optimization is also shown in Figure 6a.

The far-field diffraction efficiency is shown in Figure 6b. The black, green and red lines indicate the diffraction pattern of the ideal blazed, un-optimized and optimized metasurfaces, respectively. The input beam is RCP light. The Fresnel reflection losses of the ideal case are ignored and the corresponding intensity of the first-order diffraction beam is normalized to be 100%. The first-order diffraction efficiency of un-optimized and optimized metasurfaces are 73.4% and 87.3%, respectively. After optimization, the diffraction efficiency of the gradient surface metasurface is increased by 14% compared to the un-optimized one. For reference, the far-field diffraction of the nanofin with a thickness of 600 nm is also shown (blue line). It has been shown that the phase modulation can be 2*π* for d = 600 nm. However, the thickness is well-below the *λ*/2 thickness predicted by both FDTD and EMT. Therefore, the polarization conversion efficiency is low. Therefore, the far-field distribution for d = 600 nm shows a zero-order diffraction beam with a polarization state of RCP while the first-order diffraction beam is LCP.

## 4. Conclusions

In summary, the phase modulation ability of a dielectric PB phase metasurface, consisting of a nanofin array with arbitrary rotation angle, is theoretically analyzed. It is generally considered that the optical thickness of the unit cell of a PB-phase metasurface is *λ*/2, i.e., a half-waveplate for polarization conversion. It is found that the *λ*/2 is not essential for a full 2*π* modulation. Nevertheless, a *λ*/2 thickness is still needed for a high polarization conversion efficiency. Moreover, based on the optimized unit cell, we further designed and optimized a gradient phase metasurface. The first-order diffractive efficiency of the seven-phase-level metasurface is increased from 73.4% to 87.3% by optimizing the $\theta_{p}$ to minimize wavefront errors via the PSO method. We believe that this method can be utilized to optimize the efficiency of phase-type meta-devices, such as meta-deflectors and metalenses.

## Data Availability

The data that support the findings of this study are available from the corresponding author upon reasonable request.
